# Effect of Agraz (*Vaccinium meridionale* Swartz) on High-Density Lipoprotein Function and Inflammation in Women with Metabolic Syndrome

**DOI:** 10.3390/antiox7120185

**Published:** 2018-12-08

**Authors:** Catalina Marín-Echeverri, Christopher N. Blesso, Maria Luz Fernández, Yeisson Galvis-Pérez, Gelmy Ciro-Gómez, Vitelbina Núñez-Rangel, Juan C. Aristizábal, Jacqueline Barona-Acevedo

**Affiliations:** 1 Food and therapeutic alternatives area, Ophidism Program, School of Microbiology, Universidad de Antioquia UdeA, Calle 70 No. 52-21, Medellín 050010, Colombia; catalina.marin@udea.edu.co (C.M.-E.); yeisson.galvis@udea.edu.co (Y.G.-P.); gelmy.ciro@udea.edu.co (G.C.-G.); vitelbina.nunez@udea.edu.co (V.N.-R.); 2 Department of Nutritional Sciences, University of Connecticut, Storrs, CT 06269, USA; christopher.blesso@uconn.edu (C.N.B.); maria-luz.fernandez@uconn.edu (M.L.F.); 3 Research Group of Physiology and Biochemistry (PHYSIS), School of Nutrition and Dietetics, Universidad de Antioquia UdeA. Calle 70 No. 52-21, Medellín 050010, Colombia; juan.aristizabal@udea.edu.co

**Keywords:** Andean berry, cardiovascular risk factors, HDL dysfunction, inflammation, oxidative stress

## Abstract

Metabolic syndrome (MetS) is associated with low-grade inflammation and high-density lipoprotein (HDL) dysfunction. Polyphenol-rich foods may improve these alterations. Agraz is a fruit rich in polyphenols (mainly anthocyanins); however, there is limited information about its effects on human health. We evaluated the effects of agraz consumption as compared to placebo on HDL function and inflammation in women with MetS. Forty volunteers (25–60 years) were included in this double-blind crossover study. Women consumed agraz or placebo over 4 weeks; separated by a 4-week washout period. HDL function (apoliprotein-A1; paraoxonase 1 (PON1) activity; cholesterol efflux capacity), oxidative stress (myeloperoxidase (MPO), advanced oxidation protein products) and inflammatory markers (serum cytokines/chemokines and peripheral blood mononuclear cell nuclear factor-kB) were measured after each period. Compared to placebo, agraz consumption did not significantly change any of the biomarkers measured. Interestingly, only after agraz period there were significant positive correlations between PON1 activities and cholesterol efflux. Additionally, there were significant inverse correlations between changes in inflammatory markers and HDL function markers and positive correlations with oxidative markers. Although polyphenol-rich foods have been shown to be beneficial for certain conditions; polyphenol-rich agraz fruit consumption did not impact inflammation and HDL function in the current study of women with MetS.

## 1. Introduction

Metabolic syndrome (MetS) is a cluster of metabolic disorders shown to raise the risk of developing atherosclerotic cardiovascular diseases (CVDs) [1], which represent the leading cause of death worldwide [2]. This syndrome is associated with low-grade chronic inflammation characterized by increased C-reactive protein (CRP) [3], cytokines such as interleukin (IL)-6, tumor necrosis factor-α (TNF-α) [4], monocyte chemoattractant protein-1 (MCP-1) [5], and IL-8 [6], supporting the evidence that inflammation plays an important role in cardiovascular risk.

In addition, low high-density lipoprotein cholesterol (HDL-c) is another important component of MetS which is independently and inversely associated with cardiovascular risk [7]. However, this association seems to be complex given that high/normal HDL-c levels have been observed in people with CVD [8,9]. Disappointing results with drug therapies aiming to increase HDL-c have been reported, as some studies have not shown to prevent future cardiovascular events with increases in HDL-c [10,11]. This information suggests that other aspects of HDL should be considered.

Independently of the HDL-c level, HDL particles have shown atheroprotective roles through different functions such as reverse cholesterol transport [12], as well as anti-inflammatory [13] and anti-oxidant [14] activities. In chronic inflammatory states like MetS, dysfunctional HDL has been observed [15], characterized by low cholesterol efflux [16] and depletion of HDL-associated atheroprotective proteins including apolipoprotein A-1 (apoA-1) [17] and paraoxonase 1 (PON1), which are involved in cholesterol efflux [18,19], and antioxidant [20,21] and anti-inflammatory properties [22,23]. Likewise, myeloperoxidase (MPO), a protein able to induce oxidative modification in HDL [24] and low-density lipoproteins (LDL) [25], has been found significantly elevated in people with MetS [26]. Similarly, advanced oxidation protein products (AOPP), formed mainly from oxidation with hypochlorous acid (HOCL), generated by MPO [27], has been associated with MetS [28].

Improvements in HDL functionality are being considered and could be more effective approaches to ameliorate CVD risk. For example, several clinical studies have shown that consumption of flavonoids influence HDL functionality, through increases in HDL-c levels [29], PON1 activity [30,31], serum cholesterol efflux capacity [29,31], HDL antioxidant capacity, and reduction of HDL lipid hydroperoxides [31]. These antioxidant compounds have also shown to inhibit the activation of nuclear factor kappa B (NF-κB)—an important transcription factor that regulates inflammatory responses [32,33].

Agraz (*Vaccinium meridionale* Swartz) is a fruit rich in flavonoids mainly anthocyanins which grows in Colombia as a wild berry. This fruit has shown high antioxidant activity [34] and ex vivo cardioprotective effect [35], which have aroused interest for its potential health benefits. However, currently there are limited published studies evaluating the effects of this berry in human health. We assessed the effects of agraz consumption, compared to placebo, on HDL function and inflammation markers in women with MetS. We hypothesized that agraz consumption, compared to placebo, would improve HDL function and decrease inflammation in this group of women.

## 2. Materials and Methods

### 2.1. Study Population

Forty women (*n* = 40; 25–60 years) with MetS, according to National Cholesterol Education Program (NCEP) Adult Treatment Panel III (ATP-III) guidelines [36] were recruited from Medellin-Colombia. MetS was defined as the presence of three or more of the following risk factors: waist circumference ≥ 88 cm, triglycerides ≥ 150 mg/dL, HDL-c < 50 mg/dL, blood pressure ≥ 130/≥ 85 mmHg, and fasting plasma glucose ≥ 100 mg/dL [36]. Those who had kidney disease, heart disease, diabetes, triglycerides ≥ 500 mg/dL, fasting plasma glucose ≥ 126 mg/dL, LDL cholesterol ≥ 190 mg/dL, blood pressure > 140/90 mm Hg, consumed anti-inflammatory, lipid-lowering, hypoglycemic, and/or anti-hypertensive medications, consumed more than 20 g alcohol per day, smoked, were pregnant or planning to become pregnant, were high performance athletes, and/or consumed supplements or nutraceuticals, were excluded. This study was approved by the Human Bioethics Committee of the *Sede de Investigación Universitaria, Universidad de Antioquia* (Act No. 15-35-558-02). All participants signed the informed consent format.

### 2.2. Experimental Design

**Figure 1:**
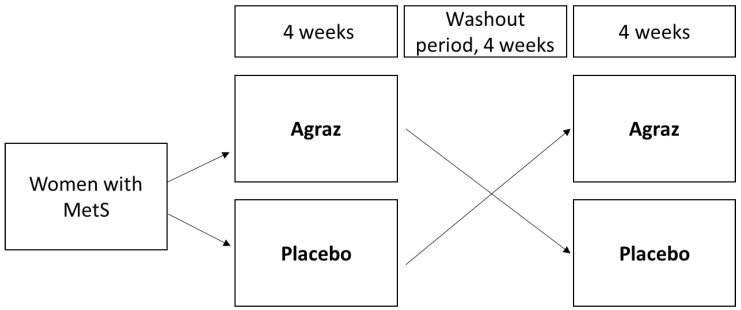
Study protocol. Women consumed agraz and placebo in a crossover design during 12 weeks (including a washout period). MetS: metabolic syndrome.

A double-blind study with a crossover design for 12 weeks was carried out. The volunteers were assigned to consume daily either agraz or placebo over 4 weeks, after which participants had a 4-week washout period, then they were allocated to the alternate treatment for additional 4 weeks (Figure 1). Freeze-dried agraz was reconstituted in 200 mL of water. The daily agraz dose was equivalent to the total phenols present in 200 g of fresh agraz (1027.97 ± 41.99 mg gallic acid equivalents (GAE)/L of agraz beverage). Placebo was designed to match the agraz beverage in terms of look, feel, taste, and macronutrients but without any polyphenols. The physico-chemical characterization, antioxidant capacity, total phenols and anthocyanin composition of agraz and placebo used in this study has been previously described [37]. During the whole study, including the washout period, volunteers were asked to abstain from consuming polyphenol-rich foods such as grapes, other berries, wine and tea or derived products. Participants registered daily consumption of the beverages, and a weekly questionnaire to assess adherence to the study to ensure they drank the beverages as indicated. When compliance was lower than 80%, participants were withdrawn from the study. Additionally, participants filled out a 7-day physical activity record and a food frequency questionnaire [38] at the beginning and end of each period to verify no changes in diet or exercise. Blood samples and data collection were obtained at the end of each consumption period.

### 2.3. Blood Collection and Peripheral Blood Mononuclear Cell (PBMC) Isolation

Blood samples were obtained after a 12-h overnight fast, from the antecubital vein using serum separator tube (yellow-topped tube) and tubes with EDTA (Vacutainer^®^, Franklin Lakes, NJ, USA). The blood collected with the yellow-topped tube was allowed to stand for 30 min, centrifuged at 2000× *g* for 10 min, and the serum frozen at −70 °C for further analysis. Whole blood collected with EDTA tubes was immediately used to isolate PBMC using Histopaqu^®^-1077 (Sigma-Aldrich, St. Louis, MO, USA).

### 2.4. Anthropometric and Blood Pressure Measurements

Waist circumference was measured at the end of a normal expiration, at the superior border of the iliac crest using a nonflexible body tape (Lufkin W606PM, Sparks, MD, USA) with an accuracy of 0.1 cm. Systolic and diastolic blood pressure were measured with an automated monitor (Omron, Healthcare, Hoffman Estates, IL, USA) on the left arm, at the heart level after at least 5 min of resting in sitting position. Two measurements were made by at least 1 min of difference.

### 2.5. Biochemical Markers

Serum glucose and lipid profile concentrations were performed by colorimetric and enzymatic methods (Siemens^®^, Washington, DC, USA) using an automatic analyzer (Dimension RxL, Siemens, Washington, DC, USA). LDL cholesterol (LDL-c) concentration was calculated using the Friedewald formula [39].

### 2.6. PON1 Arylesterase Activity

This activity was measured in serum using phenyl acetate (SigmaAldrich, St. Louis, MO, USA) as a substrate, following the methodology described by Farrell et al. [40]. Samples were diluted 400-fold in assay buffer (50 mM Tris, 1 mM CaCl_2_, pH 8.0) and processed in duplicate in a UV-compatible half-area 96-well plate (Corning Inc., Corning, NY, USA). Then, substrate buffer (3 mM phenyl acetate, 50 mM Tris, 1 mM CaCl_2_, pH 8.0) was added to each well and the reaction was finally measured at 270 nm (25 °C) every 20 seconds for 3 min using a microplate reader (Epoch Microplate UV/Vis Spec, Winooski, VT, USA). The results were expressed as kU/L using the molar extinction coefficient of phenol (0.00131 µM^−1^ cm^−1^).

### 2.7. PON1 Lactonase Activity

PON1 lactonase activity was measured in serum diluted 200-fold in sample buffer (1 mM CaCl_2_, 2.5 mM bicine, 200 mm NaCl, pH 8.3) using the method described by Millar CL et al. [41]. Each sample was added by duplicate in UV-compatible half-area 96-well plate (Corning Inc., Corning, NY, USA) and mixed with substrate buffer (0.2 mM m-cresol purple, 3 mM delta-valerolactone, pH 8.3). Finally, the reaction was measured at 577 nm (25 °C) every 20 seconds for 6 min using a microplate reader (Epoch Microplate UV/Vis Spec, Winooski, VT, USA). The results were obtained from a calibration curve with 0.1M HCl and substrate buffer, in a range of 0–115 μM.

### 2.8. Myeloperoxidase (MPO)

Serum MPO concentration was determined through an immunoenzymatic assay (MPO human ELISA (Enzyme-Linked Immuno Sorbent Assay) Kit, Cayman Chemical, Ann Arbor, MI, USA) following the manufacturer’s instructions. Briefly, standard and samples were added to a coated plate with monoclonal antibodies specific for MPO and were incubated. Horseradish Peroxidase (HRP)-labeled MPO monoclonal antibody to detect the captured MPO was added. Finally, the chromogenic substrate TMB (3,3′,5,5′-Tetramethylbenzidine) was added, incubated and the reaction was stopped with an acid solution. The yellow color formed was measured at 450 nm using a microplate reader (Multiskan Go, Thermo Scientific, Waltham, MA, USA). The intensity of the yellow color was directly proportional to the MPO concentration.

### 2.9. ApoB Precipitation

ApoB-depleted serum was obtained by precipitation of apoB-containing lipoproteins using polyethylene glycol (PEG, Pointe Scientific, INC. Ann Arbor, MI, USA), following the manufacturer’s instructions. Briefly, serum was mixed with PEG in a 1:1 ratio. After mixing 10 times by inversion, samples were incubated on ice for 20 min and centrifuged at 10,000× *g* for 10 min at 4 °C. Next, the supernatant (containing the HDL) was separated and used for the measurement of AOPP.

### 2.10. Advanced Oxidation Protein Products (AOPP)

ApoB-depleted serum was diluted in 1× Phosphate Buffered Saline-PBS in a 1:5 ratio. Then, 200 μL of each diluted sample were mixed in duplicate with 10 μL of 1.16 M potassium iodide and 20 μL of glacial acetic acid. The mixture was centrifuged at 4000 RPM for 10 min at 4 °C and the supernatant was read in duplicate at 340 nm in a UV-compatible half-area 96-well plate (Corning Inc., Corning, NY, USA). Finally, the results were compared with a calibration curve with chloramine T in a range of 0 to 100 μM. The results were expressed in units of chloramine T.

### 2.11. Cholesterol Efflux

J774 macrophages (ATCC, Manassas, VA, USA) were used to measure the cholesterol efflux capacity in apoB-depleted serum obtained after both intervention periods (agraz versus placebo). Cholesterol efflux was measured using the protocol described by Millar et al. [41] Cells were cultured on 24-well plates (0.7 × 10^5^ cells/well), with RPMI (Roswell Park Memorial Institute) media (Sigma-Aldrich, St. Louis, MO, USA), 10% fetal bovine serum (FBS; Hyclone, Logan, UT, USA) and penicillin/streptomycin (ThermoFisher Scientific, Waltham, MA, USA) and incubated at 37 °C with 5% CO_2_. After 24 h, cells were treated with RPMI media and 1% FBS, and loaded with [1,2-3H(N)]-cholesterol (Perkin Elmer, Waltham, MA, USA) and CI 976 4 mg/mL (2,2-dimethyl-N-(2,4,6-trimethoxyphenyl) dodecanamide) (Sigma-Aldrich, St. Louis, MO, USA) for 24 h in the same incubation conditions. Then, cells were treated with Cpt-cAMP 25 mg/mL (8-(4-Chlorophenylthio) adenosine 3′,5′-cyclic monophosphate) (Sigma-Aldrich, St. Louis, MO, USA) in RPMI media and 0.2% bovine serum albumin (BSA) and incubated for 16 h at 37 °C with 5% CO_2_, to promote the activation of ATP-binding cassette transporter A1 (ABCA1). After washing the cells, 2.8% of apoB-depleted serum in RPMI media with 0.2% BSA was added to the cells (in triplicate). Efflux was performed for 4 h at 37 °C with 5% CO_2_ followed by collection of cell media and cell lysates. Cell lysates were obtained by washing cells with 0.1 N NaOH and collection of the supernatant. Then, cell media and cell lysates were diluted into liquid scintillation cocktail and counted on the Beckman LS 6500 Scintillation Counter. Percent of cholesterol efflux was calculated as follows:

$$\left[\frac{3H-cholesterol\;radioactivity\;in\;media}{\left(3H-cholesterol\;radioactivity\;in\;media+3H-cholesterol\;radioactivity\;in\;cell\;lysate\right)}\right]*100$$

### 2.12. Inflammatory Markers

Serum concentration of TNF-α, MCP-1, IL-6, IL-8, and IL-1β were measured using the Human cytokine magnetic panel kit (catalog number HCYTOMAG-60K-05, Millipore Corporation, Burlington, MA, USA), using Luminex xMAP^®^ technology (Millipore Corporation, Burlington, MA, USA) and following the manufacturer’s instructions. For the NF-κB measurement, the nuclear component from the PBMC was extracted first, through a nuclear extraction kit (Abcam ab113474) according to the manufacturer´s protocol. Then, the NF-κB transcription factor was measured using the ELISA NFκB p65 transcription factor assay kit (Abcam ab133112) according to manufacturer´s instructions. Absorbances were obtained at 450 nm.

### 2.13. Statistical Analysis

Results are described based on summary measures such as mean and standard deviation (SD). The data distribution was analyze using the normality test of Shapiro Wilk. Data without normal distribution were log-transformed. Paired samples *t*-tests were conducted to analyze differences between the agraz and placebo periods. Pearson and Spearman correlations coefficient were used. All analysis were done using SPSS version 21 for Windows (SPSS, IBM Corporation, Chicago, IL, USA). Differences were considered significant at the levels * *p* < 0.05, ** *p* < 0.01, *** *p* < 0.001.

## 3. Results

### 3.1. Participant Characteristics and MetS Criteria

Forty women (47.2 ± 9.4 years old) with MetS finished the study with an adherence above 90%. In addition, there were no differences in macronutrient intake and physical activity during the whole study. Baseline MetS characteristics are shown in Table 1. The HDL-c mean for these women was 42.2 ± 6.4 mg/dL at the beginning of the study (Table 1).

### 3.2. HDL Function and Related Oxidative Markers

There were no differences in apoA-1 concentrations, PON1 activities, cholesterol efflux capacity, MPO concentration, MPO/PON1 ratio and AOPP, after comparing the end of both intervention periods (placebo versus agraz) (*p* > 0.05, Table 2).

**Figure 2:**
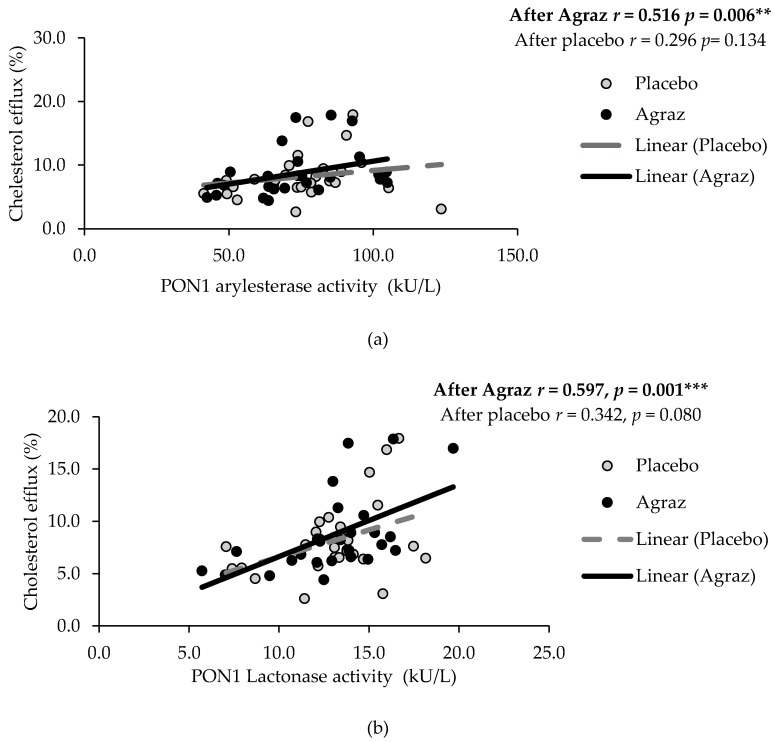
Spearman correlations between cholesterol efflux with (**a**) PON1 (paraoxonase 1) arylesterase and (**b**) lactonase activity after agraz consumption, compared to placebo. Significance * *p* < 0.05, ** *p* < 0.01, *** *p* < 0.001.

**Figure 3:**
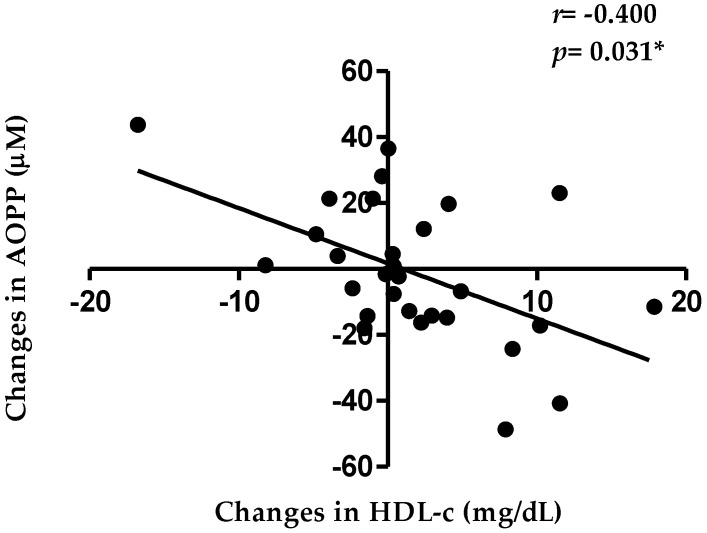
Pearson correlation between changes in high-density lipoprotein cholesterol (HDL-c) and advanced oxidation protein products (AOPP) after agraz consumption, compared to placebo. Significance * *p* < 0.05.

After agraz consumption, but not after placebo consumption, there were moderate positives correlations between cholesterol efflux capacity with PON1 arylesterase activity (*r* = 0.516, *p* = 0.006) and lactonase activity (*r* = 0.597, *p* = 0.001) (Figure 2). Likewise, there was a negative correlation between the changes in HDL-c and changes in AOPP (*r* = −0.400; *p* = 0.031) (Figure 3), a marker of oxidative stress associated with HDL.

### 3.3. Inflammatory Markers

There were no significant differences in inflammatory markers in this group of women after agraz consumption compared placebo (Table 3).

**Figure 4:**
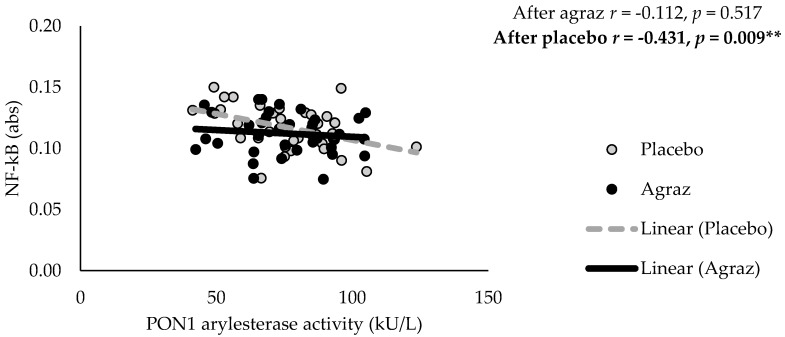
Pearson correlation between nuclear factor kappa B (NF-κB) and paraoxonase 1 (PON1) after agraz consumption, compared to placebo. Significance ** *p* < 0.01.

However, changes in inflammatory markers were inversely correlated with changes in PON1 activity and cholesterol efflux capacity. Moreover, changes in MPO and MPO/PON1 ratio were positively correlated with inflammatory markers (Table 4). Likewise, NF-κB levels were significantly and negatively correlated with PON1 arylesterase activity (*r* = −0.431, *p* = 0.009), only after placebo consumption. This correlation was not observed after agraz period (Figure 4).

## 4. Discussion

The MetS is strongly linked to low-grade chronic inflammation, oxidative stress and HDL dysfunction. In this study, we evaluated the effects of consuming a fruit (agraz) rich in polyphenols (mainly anthocyanins) on these factors, which increase cardiovascular risk.

PON1 activity was measured as a HDL function marker for its atheroprotective role. Improvements in PON1 activity have been reported following the consumption of polyphenol-rich beverages like pomegranate. An increase in PON1 activity was observed following the daily consumption of a pomegranate beverage (total phenol content of 2600 mg GAE/L of juice) for 4 weeks in 30 patients with type 2 diabetes [30]. In our study, we did not detect any significant effect on PON1 activity measures. This could be related to the lower dose provided (total phenols: 1027.97 ± 41.99 mg GAE/L of agraz beverage) compared to the above studies.

Cholesterol efflux capacity is another important marker that reflects the role of HDL in atheroprotection. Several studies have evaluated the effects of dietary interventions on this HDL-dependent process. For example, anthocyanin supplementation (80 mg of anthocyanins twice per day, Medox^®^, Sandnes, Norway) over 24 weeks showed an increase in cholesterol efflux in macrophages cultured with the serum of hypercholesterolemic subjects (55.3 ± 5.0 years) [31]. In our study, after agraz consumption, cholesterol efflux did not change compared to placebo. Similarly, one-week intervention with 250 mL of polyphenol-rich beverages (6800 ± 100 mg GAE/L of juice) in 6 healthy male subjects (25–30 years), did not significantly improve cholesterol efflux rate from cells to serum, compared to serum baseline. However, cholesterol accumulation decreased significantly in macrophages cultured in the post-treatment serum. The authors of this last study concluded these results are probably due to inhibition of cholesterol-rich lipoprotein uptake by the cells, mediated by serum-associated polyphenols [42]. 

PON1 has been shown to stimulate HDL binding and HDL-mediated macrophage cholesterol efflux via the ABCA1 transporter [18]. We found a positive correlation between PON1 activity and cholesterol efflux after agraz consumption, but not after placebo consumption, suggesting a stronger link between these HDL markers with agraz consumption. A study with anthocyanin supplementation in subjects with hypercholesterolemia demonstrated an increased in HDL-associated PON1 activity and an improvement in cholesterol efflux capacity. This suggests that modulation of PON1 activity is a potential mechanism by which anthocyanins regulate cholesterol efflux capacity [31]. This increase in PON1 activity by polyphenols could be also associated with their capacity to modulate the expression level of the PON1 gene, acting as ligands for the aryl hydrocarbon receptor -AhR [43]; or for the peroxisome proliferator-activated receptor gamma (PPAR-γ) pathway [44].

MPO is able to induce HDL dysfunction through oxidative modifications, while PON1 exerts antioxidant activities on HDL [20,21,24]. Therefore, an increase in the MPO/PON1 ratio is considered as a potential marker of HDL dysfunction [45]. We did not observe significant changes in these parameters. However, a study in 55 healthy males evaluated the consumption of a polyphenol rich-food (cocoa extract) during 4 weeks observed that MPO was significantly reduced compared to baseline; however, no changes were found compared with the placebo group [46]. In contrast to this healthy population, our volunteers had several cardiovascular risk factors, which mediate a higher state of HDL dysfunction and oxidative stress.

Additionally, MPO is the main generator of AOPP [27]–a marker of protein oxidation which contributes to atherosclerotic plaque development- [47]. AOPP was not statistically significant between treatment periods. A study with 31 healthy subjects consuming a single dose of polyphenol-rich beverage with higher total phenol content (total polyphenols 4000 mg GAE/L of beverage), reported that AOPP was reduced by 39% during the first 60 min and tended to return to baseline within 1 and 4 h after consumption [48]. From this acute study, it is evident the reduction in this marker is of short duration. In our study, blood samples were taken after 12 h of overnight fasting. Therefore, at this time of blood sampling, any acute effect on AOPP may not have been detected. However, chronic interventions have shown positive effects in this variable. For example, after 60 days of intervention with 200 mL of cranberry juice in 56 people with MetS, AOPP decreased in relationship to the baseline values [49]. Our intervention lasted 30 days, thus, longer intervention may be necessary to obtain significant changes in this variable.

AOPP is mainly generated by MPO through HOCl [27]. MPO oxidizes important proteins transported by HDL, like apoA-1, producing an oxidized apoA-1 (a type of AOPP) and affecting its capacity to remove cholesterol from cells (reverse cholesterol transport) [50]. Thus, the cholesterol-enrichment of HDL is impaired and consequently the HDL-c levels are decreased. We observed a significant negative correlation between changes in AOPP and HDL-c after agraz consumption, compared to placebo. This could be associated with the inhibitory effect of some polyphenols on MPO, through direct binding of the polyphenol molecule with the active site of this enzyme [51,52], with the corresponding decrease in AOPP formation.

Inflammation is another important component of MetS [3,4,5,6]. Various studies in both healthy populations and individuals with elevated CVD risk have found anti-inflammatory effects after chronic consumption of polyphenol-rich bilberries [53,54]. In our study, there was no differences between agraz and placebo period. Interestingly, after agraz consumption, PON1 activity (HDL function marker) had a negative correlation with inflammatory markers as compared to placebo. PON1 activity is significantly reduced in pro-inflammatory conditions such as MetS [55], possibly due to the capacity of pro-inflammatory cytokines to inhibit PON1 expression [56]. A study with polyphenols have demonstrated anti-inflammatory effects through inhibition of NF-κB activation and consequent inflammatory cascade [33]. Another study showed the capacity of polyphenols to increase PON1 expression [43], as a mechanism to improve HDL function.

Similarly, we observed significant negative correlations between cholesterol efflux capacity and pro-inflammatory markers. Although this was not a study to explore mechanisms, another (in vitro) study demonstrated that anthocyanins increased ABCA1 mRNA—a cholesterol efflux regulatory protein- and consequently increased cholesterol efflux via PPARα and LXRα, as well as inhibited the nuclear translocation of NF-κB with a reduction in pro-inflammatory protein expression [57].

## 5. Conclusions

Given that there are no previously published studies evaluating agraz consumption in people with MetS, there were no antecedents about the dose or time of intervention to be assessed with this fruit. Therefore, the daily dose used in this study was aimed to be comparable to a habitual juice consumption. The experimental design followed in this study has been employed by others evaluating polyphenol-rich fruits in people with MetS [41,58]. In conclusion, the dose of agraz used in this study during 4 weeks did not impact the HDL function, inflammatory and oxidative stress markers measured in this study, compared to placebo, in this group of women with MetS.

## Figures and Tables

**Table 1:** Baseline metabolic syndrome characteristics of women (*n* = 40).

Variables	Mean ± SD		
Age (years)	47.2	±	9.4
Waist circumference (cm)	102	±	9.2
Systolic blood pressure (mm Hg)	118.1	±	12.5
Diastolic blood pressure (mm Hg)	76.1	±	9.3
Fasting glucose (mg/dL)	94.2	±	7.3
HDL-c (mg/dL)	42.2	±	6.4
Triglycerides (mg/dL)	220.6	±	88.9

SD, standard deviation; HDL-c, high-density lipoprotein cholesterol.

**Table 2:** High-density lipoprotein (HDL) function markers after 4 weeks of agraz consumption, compared to placebo, in women with metabolic syndrome.

Variables	Placebo				Agraz				Δ Change (Agraz-Placebo) Mean ± SD			*p*
	*n*	Mean ± SD			*n*	Mean ± SD						
**HDL function markers**												
Apo A1 (mg/dL)	34	127.6	±	43.1	29	132	±	49	3.1	±	40.8	0.597
PON1 Arylesterase Activity (kU/L)	38	77.1	±	17.5	38	76.5	±	17.5	−0.7	±	8.8	0.643
PON1 Lactonase Activity (kU/L)	38	12.6	±	2.7	38	12.6	±	2.8	0.2	±	1.6	0.862
Cholesterol efflux (%)	27	8.2	±	3.6	27	8.7	±	3.8	0.5	±	2.9	0.324
**HDL-related oxidative markers**												
MPO (ng/mL)	34	177.8	±	74.6	34	175	±	72.7	−11.1	±	72	0.795
MPO/PON1 arylesterase ratio	34	2.7	±	1.6	34	2.6	±	1.3	−0.1	±	1.2	0.770
MPO/PON1 lactonase ratio	34	15.5	±	7.4	34	14.9	±	6.9	−0.7	±	6.7	0.515
AOPP (µM)	29	99.5	±	20.9	29	97.5	±	17	−2.0	±	19.8	0.703

SD, standard deviation; Apo, apolipoprotein; PON1, paraoxonase 1; MPO, myeloperoxidase; AOPP, advanced oxidation protein products. Paired *t*-test was used for the analysis. Significance *p* < 0.05.

**Table 3:** Inflammation markers after 4 weeks placebo and agraz consumption.

Variables	Placebo				Agraz				Δ Change (Agraz-Placebo) Mean ± SD			*p*
	*n*	Mean ± SD			*n*	Mean ± SD						
IL-1β (pg/mL)	37	0.8	±	0.4	37	0.8	±	0.4	0.0	±	0.2	0.748
IL-6 (pg/mL)	37	2.6	±	2.1	37	2.1	±	1.2	−0.5	±	1.5	0.271
IL-8 (pg/mL)	37	12.6	±	5.6	37	12.1	±	5.5	−0.3	±	2.6	0.322
MCP-1 (pg/mL)	38	251	±	103	38	248.3	±	106.6	−2.6	±	47.3	0.479
TNF-α (pg/mL)	38	4.7	±	1.8	38	4.6	±	1.5	−0.1	±	0.8	0.257
NF-κB (abs)	38	0.1	±	0.02	38	0.1	±	0.02	0.0	±	0.02	0.290

SD, standard deviation; TNF-α, tumor necrosis factor-alpha; IL, interleukin; MCP-1, monocyte chemoattractant protein-1; NF-κB, nuclear factor kappa B. Paired *t*-test was used for the analysis. Significance *p* < 0.05.

**Table 4:** Correlations^1^ between changes in high-density lipoprotein (HDL) function and inflammation markers after agraz consumption, compared to placebo, in women with metabolic syndrome.

Changes in Variables	IL-1β (pg/mL)	IL-6 (pg/mL)	IL-8 (pg/mL)	MCP-1 (pg/mL)	TNF-α (pg/mL)
Apo A1 (mg/dL)	0.151	0,022	0.056	−0.087	0.030
PON1 Arylesterase Activity (kU/L)	0.215	−0.273	−0.106	−0.060	−0.012
PON1 Lactonase Activity (kU/L)	0.060	−0.390*	−0.169	0.145	−0.213
MPO (ng/mL)	0.102	0.707 ***	0.338	0.413 *	0.196
MPO/PON1 arylesterase ratio	0.097	0.682 ***	0.349	0.393 *	0.229
MPO/PON1 lactonase ratio	0.099	0.701 ***	0.323	0.295	0.202
AOPP (µM)	0.098	0.080	−0.228	0.170	−0.087
Cholesterol efflux (%)	−0.594 ***	−0.283	−0.128	−0.148	−0.496 **

^1^ Pearson and Spearman correlation coefficients. Significance at * *p* < 0.05, ** *p* < 0.01, *** *p* < 0.001. Apo, apolipoprotein; PON1, paraoxonase 1; MPO, myeloperoxidase; AOPP, advanced oxidation protein products; TNF-α, tumor necrosis factor-alpha; IL, interleukin; MCP-1, monocyte chemoattractant protein-1.
